# Development of a Dual Drug-Loaded, Surfactant-Stabilized Contrast Agent Containing Oxygen

**DOI:** 10.3390/polym14081568

**Published:** 2022-04-12

**Authors:** Raj Patel, Quezia Lacerda, Brian E. Oeffinger, John R. Eisenbrey, Ankit K. Rochani, Gagan Kaushal, Corinne E. Wessner, Margaret A. Wheatley

**Affiliations:** 1 School of Biomedical Engineering Science and Health Systems, Drexel University, Philadelphia, PA 19104, USA; rsp65@dragons.drexel.edu (R.P.); quezia.lacerda@jefferson.edu (Q.L.); brian.e.oeffinger@drexel.edu (B.E.O.); 2 Department of Radiology, Thomas Jefferson University, Philadelphia, PA 19107, USA; john.eisenbrey@jefferson.edu (J.R.E.); cw3273@drexel.edu (C.E.W.); 3 Department of Pharmaceutical Sciences, Thomas Jefferson University, Philadelphia, PA 19107, USA; ankit.rochani@jefferson.edu (A.K.R.); gagan.kaushal@jefferson.edu (G.K.)

**Keywords:** ultrasound contrast agent, microbubbles, theranostic agents, ultrasound-triggered drug delivery, dual drug loading, surfactant, oxygen delivery

## Abstract

Co-delivery of cancer therapeutics improves efficacy and encourages synergy, but delivery faces challenges, including multidrug resistance and spatiotemporal distribution of therapeutics. To address these, we added paclitaxel to previously developed acoustically labile, oxygen-core, surfactant-stabilized microbubbles encapsulating lonidamine, with the aim of developing an agent containing both a therapeutic gas and two drugs acting in combination. Upon comparison of unloaded, single-loaded, and dual-loaded microbubbles, size (~1.7 µm) and yield (~2 × 10^9^ microbubbles/mL) (~1.7) were not statistically different, nor were acoustic properties (maximum in vitro enhancements roughly 18 dB, in vitro enhancements roughly 18 dB). Both drugs encapsulated above required doses calculated for head and neck squamous cell carcinoma, the cancer of choice. Interestingly, paclitaxel encapsulation efficiency increased from 1.66% to 3.48% when lonidamine was included. During preparation, the combination of single drug-loaded micelles gave higher encapsulation (µg drug/g microbubbles) than micelles loaded with either drug alone (lonidamine, 104.85 ± 22.87 vs. 87.54 ± 16.41), paclitaxel (187.35 ± 8.38 vs. 136.51 ± 30.66). In vivo intravenous microbubbles produced prompt ultrasound enhancement within tumors lasting 3–5 min, indicating penetration into tumor vasculature. The ability to locally destroy the microbubble within the tumor vasculature was confirmed using a series of higher intensity ultrasound pulses. This ability to locally destroy microbubbles shows therapeutic promise that warrants further investigation.

## 1. Introduction

Multidrug resistance (MDR) restricts chemotherapeutic power and presents a major impediment in the treatment of cancer, with MDR believed to be the cause of treatment failure in over 90% of patients with metastatic disease [1]. Classical MDR results from an overexpressed, energy-dependent, and unidirectional drug efflux pump that is cell-membrane bound and composed of a transmembrane glycoprotein (P-gp) [2,3]. We are interested in head and neck cancer, and various studies have implicated P-gp expression in MDR in these cancers [4,5,6,7]. Multiple types of non-ionic surfactants and polymers have been shown to inhibit the P-gp efflux pump, including Cremophor EL, D-alpha-tocopherol-poly (ethylene glycol 1000) succinate (TPGS), Tween80, and various chitosan derivatives. This inhibition can in turn result in increased intracellular concentrations of chemotherapeutics such as paclitaxel (PTX), and this results in enhanced therapeutic efficacy against MDR tumors [2]. Therapeutics can also target MDR and the P-gp efflux pump by halting intracellular adenosine triphosphate (ATP) production capabilities and promoting pro-apoptotic factors. These include colchicines, 3-bromopyruvate, and lonidamine (LND). Lonidamine, in particular, has been shown to be able to inhibit the hexokinase enzyme that is pivotal to mitochondrial function, making it attractive in therapies that treat multiple types of cancer [2]. Importantly, LND is also able to sensitize tumors to radiotherapy and photodynamic therapy [8]. Huang et al. point to the unique characteristics of tumors’ energy metabolism and the Warburg effect and hence suggest its advantages of selectivity and lack of overlapping adverse effects [9].

A frequent precursor to the development of MDR is hypoxia [10], which is prevalent in up to 70% of head and neck cancers [11,12]. Hypoxia can arise from a disruption of tumor microcirculation, giving rise to large diffusion distances (>70 μm) that oxygen must travel before it reaches the cell, a phenomenon known as chronic or diffusion-limited hypoxia. Hypoxia is especially problematic as it allows solid tumors to become resistant to radiotherapy and chemotherapy and hastens tumor progression [12]. Oxygen is a powerful chemical radiosensitizer and plays a vital role in permanently securing the DNA damage caused by radiotherapy. The radiation dose needs to be increased almost three-fold in the absence of oxygen compared to normal levels just to keep equivalent efficacy. Hypoxia may increase radiation resistance by increasing the levels of heat shock proteins and decreasing apoptotic potential, both of which have been linked to radiation resistance and proteome changes that strongly impact tumor propagation [13,14]. Fortunately, very little oxygen is needed to sensitize hypoxic tumors to radiotherapy [15]. Hypoxia also interferes with chemotherapy due to indirect effects associated with increased glycolysis and extracellular acidosis [12]. Regulation of hypoxia in the tumor also plays a key role in other therapies, for example in photodynamic therapy, which relies on the generation of reactive oxygen species (ROS) to kill cancer cells, a mechanism that is severely restricted by hypoxia [16].

Combinatorial therapy is especially effective in cancer as it can target different key pathways in a tumor and often results in lowering the required therapeutic dosage of a given drug [17,18]. One such combination therapy for MDR involves the dual delivery of PTX and LND. The combination of LND’s role in intracellular ATP suppression and P-gp inhibition leads to increased accumulation of intracellular PTX, which overall leads to the induction of apoptosis in tumor cells [2]. Multimodal therapies that combine different modes of therapy such as chemotherapy and radiotherapy (known as chemoradiotherapy) [19,20] are often highly effective. These result in “super-additive” effects where the combined therapy is much stronger and more efficacious than a single type [21]. For example, Puiu et al. even suggested the dual use of magnetically targetable SPIONs based on magnetite (Fe_3_O_4_) nanoparticles’ surfaces modified with β-cyclodextrin (CD) and PTX-guest-host inclusion complexes [22].

Several groups have investigated the dual delivery of PTX and LND as a combination therapy to target and reverse MDR in cancers [2,23]. However, to our knowledge, addressing the additional problem of relieving hypoxia has not also been considered.

**Figure 1:**
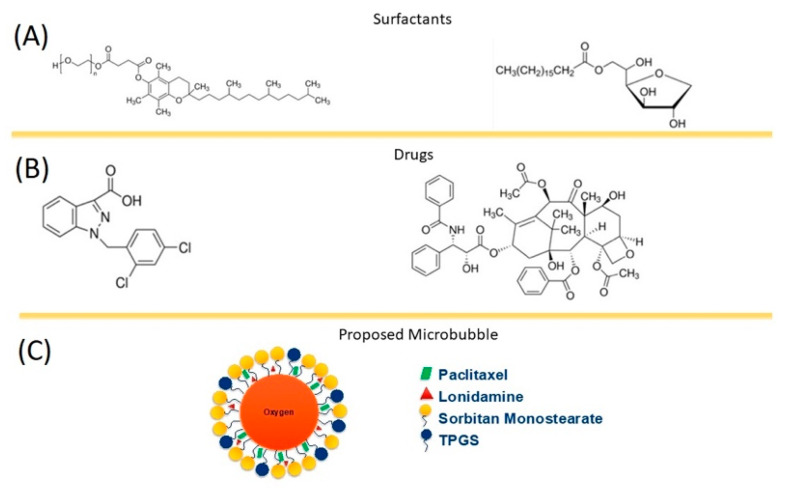
Microbubble shell component. (**A**) Surfactants, (**B**) Drugs, (**C**) Proposed model.

Previously, our group designed an oxygen core, surfactant-stabilized microbubble named SE61_O2_ which is composed of a sorbitan monostearate and TPGS shell (Figure 1). Toxicity studies using microbubbles with this and very similar shell chemistry have demonstrated the agent is non-toxic and well tolerated [24,25]. This microbubble acts as an ultrasound contrast agent for enhanced imaging of tumors, as well as a delivery vehicle of oxygen for the sensitization of hypoxic breast tumors for radiotherapy [26].

We have shown that SE61_O2_ microbubbles increase local oxygen levels when insonated with a 4-megahertz ultrasound at a 3.6 MPa peak-to-peak pressure [24]. Further studies on mouse models with MDA-MB-231 breast cancer xenografts showed increased pO_2_ levels in tumors when receiving intravenous SE61_O2_ with ultrasound at 4.2 MHz, a peak negative pressure of 2.5 MPA, and a pulse repetition frequency of 38 Hz. Increased radiosensitivity was demonstrated when 5 Gy of radiation followed, post insonation [26]. In addition to this, our in vivo studies on murine metastatic breast cancer models in the brain have shown decreased tumor volume in mice receiving oxygen core SE61_O2_ microbubbles followed by 10 Gy of radiation [27]. We recently loaded the microbubble with LND to further increase the duration of oxygen present in the tumor post-delivery through inhibition of tumor mitochondrial respiration [28]. 

The aim of the current study is to explore the possibility of further leveraging the LND-SE61_O2_ microbubble by including PTX, thus expanding the capabilities to include dual drug encapsulation, for example in the elimination of MDR [29]. An added rationale is that the oxygen core of the microbubble will allow for local gas delivery to the hypoxic tumor site, reducing hypoxia-associated drug resistance and enhancing its susceptibility to radiotherapy [26,30]. The LND will inhibit the cancer cells’ metabolic functions, which will further prolong oxygen presence in cells for radiotherapy and inhibit the ATP-dependent drug efflux pumps, which will in turn reduce the tumor’s MDR to chemotherapy [26,28]. The introduction of PTX will promote apoptosis for cancer cell death and arrest cells in G2/M phase, further sensitizing the tumor to irradiation [31]. TPGS in the wall of SE61_O2_, while acting as a microbubble stabilizer, can participate in providing a hydrophobic space for drug encapsulation and has the added potential advantage of further attacking MDR through its ability to inhibit the P-gp efflux pump [32]. 

We posit that the existing LND-loaded SE61_O2_ microbubble investigated to sensitize hypoxic tumors for radiotherapy could be similarly leveraged for combination and multimodal cancer therapy through the inclusion of PTX in its shell. Previous studies of our family of surfactant-stabilized bubbles suggest that the microbubbles are stabilized by a single surfactant layer, with the hydrophobic tails facing inwards, initially towards a hydrophobic perfluorocarbon (PFC) gas [33]. We propose that a second hydrophobic drug could be accommodated in the hydrophobic tail section of this monolayer. This study acts as a proof of principle for the application of this design in the dual-drug loading of the SE61_O2_ microbubble. 

## 2. Methods

### 2.1. Materials

The drug LND was purchased from Millipore Sigma (Allentown, PA, USA) and PTX from LC Laboratories (Woburn, MA, USA). Materials for microbubble preparation included TPGS, (Millipore Sigma, Allentown, PA, USA), sorbitan monostearate (Montane 60 PHA Premium), a gift from Seppic (Fairfield, NJ, USA), octafluropropane (OFP) Specialty Gases of America (Reno, NV, USA), oxygen gas from Airgas (Radnor, PA, USA), and passed through a sterile 0.22-micrometer Nalgene filter (Nalge Nunc International, Rochester, NY, USA) at an initial flow rate of 50 mL/min for 5–10 s then 20 mL/min for 1 min prior to use. All other chemicals were from Millipore Sigma (Allentown, PA, USA) and used as received. 

### 2.2. Unloaded SE61_O2_ Microbubble Fabrication 

Unloaded microbubbles were prepared by a previously described method [34]. Briefly, micelles were formed by heating 1.288 g of TPGS dissolved in 25 mL of 37 °C phosphate-buffered saline (PBS) and placed on a stir plate for ~30 min. In parallel, 1.464 g of sorbitan monostearate and 1.5 g of NaCl in 25 mL PBS solution were autoclaved (Yamato Scientific Co., Ltd., Tokyo, Japan) for 35 min at 125 °C to aid in dispersion. Initial micelle formation was found in a previous study to generate over double the microbubble yield [34]. The autoclaved solution was immediately added to the TPGS micelle solution and allowed to cool to room temperature under continuous mixing. The mixture was then placed on an ice bath and purged with PFC gas for 1 min. The mixture was then sonicated under PFC purging, using a 0.5 inch probe horn (Misonix, Inc., Farmingdale, NY, USA), at 20 kHz for 3 min at 110 W to generate the microbubbles. The microbubble mixture was then placed in a 250 mL separatory funnel for gravity separation, discarding the bottom layer containing unused surfactant, and the top foam layer, and retaining the middle, microbubble layer. The microbubbles were washed a total of three times over a 4–5-h period, in increments of 1–1.5 h using 50 mL of 4 °C PBS in each wash. After the last wash, microbubbles were collected and diluted in a 1:1 (*v*/*v*) ratio with 10% (*w*/*v*) glucose to provide lyoprotection. The microbubbles were then transferred in 2-milliliter aliquots to 10-milliliter lyophilization vials (Type I AMB Glass, Duran Wheaton Kimble, Millville, NJ, USA), capped with rubber stoppers (Duran Wheaton Kimble, Millville, NJ, USA), and placed in an ice bath consisting of a 1:1 (*v*/*v*) water-propylene glycol (Haake D1 and G, Bacchus Marsh, Australia) at −20 °C. The microbubbles were gently shaken until frozen. The vials were then placed on a specially designed pre-chilled shelf (−20 °C) for at least 2 h. Lyophilization was performed using a Virtis Benchtop freeze dryer 29 (Gardiner, NY, USA) for 20 h under a condenser temperature of −70 °C below 300 μbar pressure. The caps on the vials were loosely sealed to the first groove during this process. The dried microbubbles were sealed under a vacuum at the end of the cycle by depressing the stoppers prior to venting using a piston. They were then removed and finally sealed around the stopper with parafilm and stored at −20 °C until use.

### 2.3. Single-Drug-Loaded SE61_O2_ Microbubble Fabrication

The quantity of drugs used in encapsulation was based on previous experience in our lab and the calculation of estimated minimum drug encapsulation requirements at the site.

*Estimation of minimum drug loading*: For an assumed tumor volume of 65 mm^3^, maximum injection volume in mice is 0.1 mL at an average microbubble concentration of 3.2 × 10^9^/mL, and 20% of microbubbles are destroyed at the tumor site after multiple circulatory passes [28,35]. *Lonidamine metric* is based on a minimum effective LND dose in tissue culture of 3 μM; this translates to 3.13 µg LND/mL microbubble [36]. *Paclitaxel Metric* is based on an effective dose of 1 µM in tissue culture, resulting in a desired minimum dose of 2.77 µg PTX/mL microbubble [37,38]. In preparations, 3.9 mg LND and/or 5 mg PTX were added to each batch. For single-drug loading, 1.288 g of TPGS was dissolved in 25 mL of 37 °C 1 × PBS for a short period (~10 min), after which the drug was added to the mixture and allowed to incubate and establish equilibrium for 48 h at 37 °C. 

### 2.4. Dual-Drug-Loaded SE61_O2_ Microbubble Fabrication

**Figure 2:**
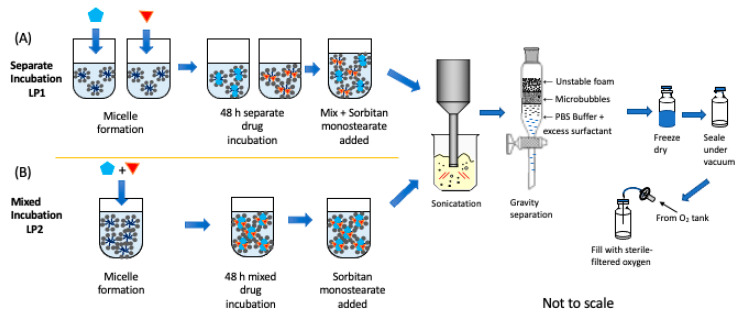
Process for making drug-loaded SE61_O2_. (**A**) Individual drug-loaded micelles (LP1), (**B**) Micelles containing two drugs (LP2). Turquoise pentagon represents paclitaxel, red triangle represents lonidamine, and grey represents surfactants. Not to scale.

For dual loading, two different approaches were explored to more clearly identify any changes/pitfalls that adding a second drug may have, such as competition for space in the shell or exclusion between drugs. The first method (LP1) consisted of making separate drug-loaded TPGS micelles for each drug before adding both to the autoclaved sorbitan solution. In this case, separate TPGS solutions were made, dissolving 0.644 g of TPGS with 12.5 mL of 37 °C PBS (to keep final TPGS concentrations consistent); 0.5 mg of PTX was added into one mixture and 3.9 mg of LND into the other, and both were allowed to incubate separately at 37 °C for 48 h before being mixed with the autoclaved sorbitan monostearate solution before the microbubble fabrication stage as above. The second method (LP2) followed similar methods but combined drugs for incubation in TPGS micelles for 48 h before microbubble fabrication, thus first forming mixed-drug micelles. Following these two distinct methods, any drug interaction during the micellation step could be identified while preserving the advantages of entrapping the drug in TPGS micelles prior to the sonication step, a method that we have identified previously as producing a doubling of microbubble yields [34]. The entire process is outlined in Figure 2.

For all analyses, each preparative run was conducted three times, analyses of subsequent samples were performed in triplicate, and the results were reported in a standard format as the mean ± standard deviation, n = 3.

### 2.5. SE61_O2_ Microbubble Size Distribution

Bubbles were counted and sized using an Entegris AccuSizer^®^ system (Billercia, MA, USA) using a diameter range between 0.9–18 μm. Measurements below 0.9 μm were considered to be residual surfactant particles since samples were taken from vials in which the bubbles had been allowed to decay and which demonstrated no measurable echogenicity produced a matching size distribution below 0.9 µm. Reconstituted bubble samples were diluted by a factor of 100 in a 1:99 (*v*/*v*) ratio, adding 10 μL of microbubbles to 990 μL of deionized water (ACS reagent, ASTM Type I). Samples (volume of 20 μL) were then injected into an automatic dilution chamber containing filtered DI water. Measurements were done using an LE sensor with a range of 0.5 to 200 µm.

### 2.6. Light Microscopy

An Olympus 1X71 microscope (Olympus Corporation, Tokyo, Japan) was used to image the microbubbles and assess if the dual-drug loading caused any observable visual changes to their general characteristics. Microbubbles were diluted in a 1:4 (*v*/*v*) ratio with DI water before imaging. Microbubble images were processed using the Olympus CellSens Standard software (Olympus Corporation, Tokyo, Japan).

### 2.7. In Vitro Acoustic Characterization

Lyophilized samples were charged with oxygen that was passed through a 0.2 μm sterile filter at around 50 mL/min for 10 s. The microbubbles were then reconstituted in 2 mL of 0.5×PBS for acoustic characterization using a 5-megahertz ultrasound transducer (part no. V309, Olympus, Waltham, MA, USA) focused inside a custom-built 50-milliliter acrylic acoustic sample chamber submerged in a 10-gallon tank filled with water at 37 °C, following a previously published protocol [24]. The transducer had a focal length of 49.3 mm, diameter of 12.7 mm, center frequency of 5.15 MHz, peak frequency of 5.36 MHz, and a −6 dB bandwidth of 82.46%. Pressure amplitudes were generated with a pulse repetition frequency of 100 Hz at energy level 1 using a parametric pulse/receiver (model 5072PR). This results in a peak negative pressure of 0.25 MPa and a peak positive pressure of 0.69 MPa. Signals were amplified by 40 dB and read using a LeCroy 9350A digital oscilloscope (Chestnut Ridge, NY, USA). For acoustic enhancement, increasing volumes of microbubbles were tested, replacing the PBS inside the chamber between each run and graphing dB returned to the transducer against dose. 

### 2.8. Flow Phantom Analysis

#### 2.8.1. Imaging

Echogenicity and microbubble destruction were also assessed separately using an in vitro closed-loop flow phantom setup (model 524, ATS Laboratories, Bridgeport, CT, USA) and clinical scanner (S3000 Helx scanner, Siemens Healthineers, Mountain View, CA, USA, with a 9L4 probe) at room temperature as previously described [39]. Samples of LP1-type bubbles (drugs initially in separate micelles) were chosen since at the time of analysis these were shown to have the highest drug encapsulation and would be taken on to future in vivo experiments. Briefly, a known concentration of roughly 1.0 × 10^7^ microbubbles in 800 mL PBS was circulated at room temperature through the tissue-mimicking flow phantom, passing through a 6-millimeter diameter vessel embedded in the device using a peristaltic pump at 350 mL/min. Imaging of the microbubbles flowing through the embedded vessel was performed in cadence pulse sequencing mode every 30 s for 20 min (n = 3, for each formulation).

#### 2.8.2. Stability Curves

The stability of the microbubbles in a clinically relevant ultrasound beam was determined by taking an image from the flow phantom every 30 s for 10 min. A region of interest (ROI) was chosen on the contrast mode image and the mean enhancement measured within that region and determined using ImageJ. This value, normalized to allow for microbubble concentration differences between samples, was used in equation 1 to determine the enhancement (dB) at each time point and plotted against time.

(1) $$\mathrm{dB}=20\log_{10}\frac{E}{E_{0}}$$

where

*E* = Mean enhancement returned to transducer

*E*_0_ = Baseline level enhancement.

### 2.9. Drug Loading Quantification

Quantification of PTX and LND was performed at Thomas Jefferson University. Samples were analyzed separately using two high-performance liquid chromatography (HPLC) methods. Drug-loaded microbubbles were extracted in 4 mL methanol and filtered using a 0.45-micrometer-syringe filter; 1 mL of extracted samples was analyzed using HPLC.

Paclitaxel samples were analyzed on a Waters^®^ HPLC Alliance 2695 separations module system using a Phenomenex Luna 5 μm 100 Å, 100 × 4.6 mm column. Detection was performed at 277 nm using a Waters^®^ 2998 photodiode array detector (Milford, MA, USA). The HPLC solvent consisted of a 30:70 (*v*/*v*) water/acetonitrile (ACN) ratio at a 0.5 mL/min flow rate. The total runtime was 5 min with a sample injection volume of 10 µL. 

Lonidamine quantification was carried out using a Thermo Scientific, Dionex Ultimate 3000 UHPLC system (Waltham, MA, USA). Samples were analyzed using an Xbridge BEH shield reverse phase C18 column (2.5 μm, 4.6 × 75 mm) and solvent ratio 50:50 water (with 0.1% formic acid [FA]) and ACN (with 0.1% FA) at a flow rate of 0.350 mL/min [40]. Detection was performed using a UV-visible spectrometer at 230 nm. The column temperature was maintained at 30 °C for analysis of samples. For LND, the total run time was 20 min and the sample injection volume was 10 µL.

Encapsulation efficiencies of the drugs were calculated using the following Equation (2):

(2) $$Encapsulation\;Efficiency=\frac{Mass\;of\;Drug\;in\;MB\;\left(\mathrm{mg}\right)}{Initial\;Mass\;of\;Drug\;Aded\;\left(\mathrm{mg}\right)}\times 100\%\;$$

### 2.10. In Vivo Studies

All animal work was performed at Thomas Jefferson University under a protocol approved by the local institutional animal care and use committee in accordance with American Association for Laboratory Animal Science guidelines. To demonstrate initial tolerability and the ability to detect and locally destroy dual-loaded microbubbles in vivo, a brief series of imaging experiments were performed in athymic nude mice (two male, one female; The Jackson Laboratory, Bar Harbor, ME, USA). Tumors were generated via subcutaneous injection of 10^6^ CAL27 oral human squamous cell carcinoma cells (ATTC, Manassas, VA, USA) with 50 µL of Matrigel (Sigma) into the right hindlimb. Tumors were monitored until reaching a total volume of approximately 150–500 mm^3^ before being used for imaging experiments. Prior to imaging, animals were anesthetized with a mixture of ketamine and acepromazine, and body temperature was maintained using a 37 °C heating pad.

Dual-loaded microbubbles made by the LP1 method—with drugs initially in separate micelles—were suspended in room-temperature PBS as described above. A total injection of 0.1 mL was then administered via a 24-gauge angiocatheter in the tail vein followed by 0.05 mL saline flush. Continuous imaging in dual B-mode/cadence pulse-sequencing contrast mode was acquired using a 10L4 transducer and Acuson Sequoia (Siemens Healthineers, MountView, CA, USA). Imaging was acquired at a depth of 3 cm and focal zone of 1 cm. Low mechanical index (MI; 0.12) imaging was used to obtain non-destructive visualization of microbubble perfusion into the tumor. Following peak enhancement, a destructive pulse (4 s at MI = 1.4) was initiated to destroy microbubbles within the tumor followed by 10 s of low-MI imaging to visualize reperfusion. This process was repeated for the duration of contrast enhancement (approximately 3–4 min). Animals were then monitored for at least 30 min for acute toxicity.

### 2.11. Statistical Analysis

All data are presented as a standard deviation about the mean. Acoustical data were measured from three microbubble lots, with each repeated three times (n = 3). Bubble counts and size data were obtained from one lot with each repeated three times (n = 3). Microbubble average size and drug loading values with standard deviations were calculated using Excel (Microsoft Office 365 Plus, Microsoft Corporation, Redmond, WA, USA). Statistical significance for each type of drug loading between dual loading conditions was determined using a one-way ANOVA with Tukey post hoc tests. Significance in acoustical properties and bubble populations between drug-loading conditions was determined using a one-factor MANOVA (Wilks’ Lambda). A significance level of α = 0.05 was used and tests were run using the software Statistical Package for the Social Sciences (SPSS, Chicago, IL, USA).

## 3. Results and Discussion

### 3.1. Incorporation of a Single Drug 

Single drug (PTX or LND) loading on the SE61 microbubble was initially analyzed to serve as a baseline for the dual-loaded microbubbles. Both LND and PTX were encapsulated as described, initially mixing the drug into a TPGS 0.052% (*w*/*v*) solution for 48 h, creating drug-loaded micelles before adding to the autoclaved sorbitan monostearate solution. This efficiently created a final mixture with a 0.026% (*w*/*v*) TPGS and 0.029% (*w*/*v*) sorbitan monostearate concentration that was then sonicated for microbubble fabrication. During both these stages, the TPGS concentration remained above its critical micelle concentration (CMC) of 0.02% (*w*/*w*), ensuring micelles were present and that the drug was ideally encapsulated within them. 

#### 3.1.1. SE61_O2_ Microbubble Size Populations

Despite differences in chemical structure, molecular weight (LND 321, PTX 853.9), and hydrophobicity (LND log P = 3.9–4., PTX log P = 7.4), separate incorporation of these drugs into the SE61_O2_ microbubbles did not have a significant effect on microbubble size population as measured by mean bubble size and total concentration (*p* = 0.86) (Table 1). Representative size distribution profiles are given in the Supplementary Materials (Figure S1) and show all groups had similar distributions. All microbubble groups had an average diameter well below the requirement of <6 μm to pass through the vasculature.

The ease of insertion was not surprising since both drugs are found to partition into lipid monolayers [23,41]. All preparations were within the size range of less than 5 µm, required for free passage through the pulmonary bed. Bubble size measurements were substantiated by light microscopy, presented in Supplementary Materials (Figures S2 and S3). This confirmation is particularly relevant when measuring buoyant particles that could influence methods of measurement dependent on Brownian motion, such as dynamic light scattering; however, with our use of the Entegris AccuSizer^®^, which depends on single particle optical sizing technology; the results were not subject to these buoyancy effects.

#### 3.1.2. In Vitro Acoustic Characterization

**Figure 3:**
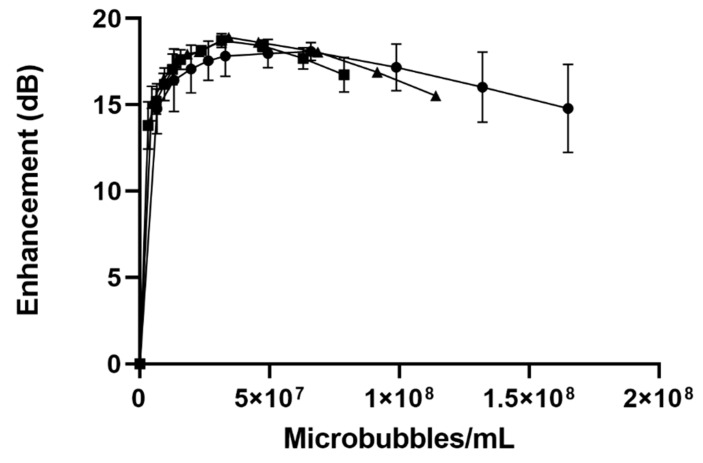
Effect of drug loading on dose-response curves (plotted as microbubbles/mL) for unloaded (-●-), lonidamine (-◾-), and paclitaxel-loaded (-▲-) SE61_O2_ microbubbles.

Our in vitro tank testing of acoustic microbubbles served as an excellent method of investigating differences in acoustic behavior among different preparations. While all measures were taken to ensure we came close to in vivo conditions (temperature, pH, and salt content of suspending medium; continuous stirring), these measurements are best used for comparison purposes, although we have shown in the past that they closely mirror in vivo measurements [42]. We considered two parameters—maximum dB achieved, and the dose at which this maximum is obtained when evaluating acoustical properties. As with size and bubble count, the measured acoustics between unloaded, LND- and PTX-SE61_O2_ microbubbles did not show any significant differences (*p* = 0.40) in acoustical properties (Figure 3).

The unloaded microbubbles had a maximum enhancement of 18.10 ± 0.52 dB, the LND-loaded microbubbles a maximum enhancement of 18.7 ± 0.41 dB, and the PTX microbubbles a maximum enhancement of 18.9 ± 0.18 dB at similar doses (Table 1). The echogenicity of a microbubble, and its resultant enhancement, are related to the backscatter area and resonance frequency. These variables are in turn mainly influenced by the difference between microbubble and medium compressibility, density, and microbubble radius to the sixth power. Drug loading within the microbubble shell is thought to influence the shell elasticity parameter, which is only a small part of the microbubble resonance, and the overall acoustic response of the microbubble. The decreasing enhancement observed at higher doses can be attributed to acoustical shadowing. 

#### 3.1.3. Drug Loading Quantification

Drug loading (LND analyzed by HPLC-MS and PTX by HPLC-UV), is tabulated in Table 2. Encapsulation efficiency was low, in keeping with the reality of the limited space available to accommodate drugs in a shell [43]. On a dose basis, the two drugs were accommodated at roughly the same amount, 4.47 ± 0.97 μg PTX/mL microbubble, and 4.19 ± 0.75 μg LND/mL microbubble. The differences were more apparent on a weight-by-weight basis (μg drug/g microbubble), reflecting the somewhat higher bubble concentration of the PTX-microbubble (2.29 ± 0.50 × 10^9^ microbubble/mL compared to LND-microbubble 1.57 ± 0.62 × 10^9^ microbubble/mL), and on a molar basis reflecting the different molecular weights.

Despite the low encapsulation efficiencies, both drugs were present at well over the calculated minimum required doses. For LND, this was 3.13 µg LND/mL microbubble. Similar calculations (outlined in the introduction) for PTX, based on an effective dose of 1 µM in tissue culture, resulted in the desired minimum dose of 2.77 µg PTX/mL microbubble.

### 3.2. Dual-Drug LND-and-PTX-Loaded SE61_O2_

Due to the different molecular weights, sizes, and hydrophilicities of LND and PTX causing concerns that encapsulating the two drugs together in the same micelle prior to sonication might result in disproportional drug loading, the investigation used both single (LP1), and dual (LP2), loaded micelles during fabrication. 

#### 3.2.1. Microbubble Size Populations for Dual Drug Loaded SE61_O2_

Microbubble diameters and concentrations of dual drug loading (two methods) are compared in Table 3. No significant effect (one-factor MANOVA, *p* = 0.136) was found among loading conditions and the bubble size population (mean diameter and total microbubble concentration) after lyophilization. Like single loaded, all microbubble groups had an average diameter well below the requirement of <6 μm. Light microscopy confirmed these results and representative images are supplied in the Supplementary Materials (Figure S2). The size distribution profiles are also given in the Supplementary Materials together with those of unloaded and single-loaded bubbles (Figure S1).

#### 3.2.2. In Vitro Acoustic Characterization

**Figure 4:**
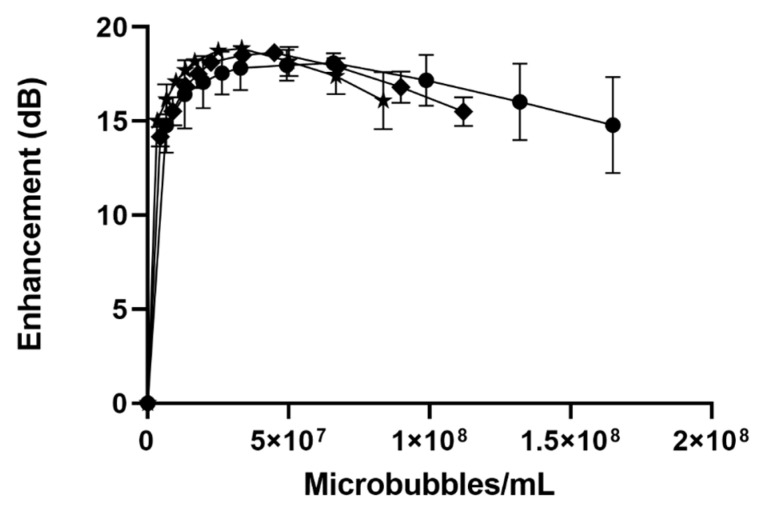
Effect of dual drug loading method on dose-response (plotted as microbubble/mL) curves for unloaded (-●-), LP1 (-◆-), and LP2 (-✮-) SE61_O2_ microbubbles.

The impact of the drug loading method on acoustic behavior can be seen in Figure 4. Both methods yielded microbubbles with a robust, dose-dependent acoustic response, and exhibited shadowing at higher doses. For dual loaded SE61_O2_ microbubbles, the maximum enhancement that was achieved was 18.63 ± 0.05 dB for LP1 (separate micelle incubation) compared with 18.8 ± 0.24 dB for Lp2, comparing favorably with unloaded bubbles (18.10 ± 0.52), as shown in Table 3. 

There was no significant effect (*p* = 0.318) of dual drug loading on acoustical properties using max enhancement and dose (one-factor MANOVA) when compared to single-drug and no-drug MBs. These data can be compared favorably with the maximum enhancement of 18.10 ± 0.52 dB for unloaded microbubbles, as reported above.

#### 3.2.3. Dual Drug Loading Quantification

##### Effect of Method on Lonidamine Encapsulation

The final drug composition in the microbubbles was analyzed to assess the suitability to become a carrier for multiple drugs and the impact, if any, of the method of preparation on the final drug compositions. As reported above, when encapsulating LND alone in the microbubbles, we have encapsulated 4.19 ± 0.75 µg LND/mL microbubble, representing an encapsulation efficiency of 1.54%. We noticed an increase in LND loading compared to LND alone when using separate drug-loaded micelles (LP1) (Table 4); however, no significant difference (*p* = 0.09) in loading among the three groups was observed. The result strongly suggests that co-loading of PTX with LND by either of the two methods did not deleteriously interfere with the LND loading. In addition, the calculated minimum-required drug concentration of 3.13 µg LND/mL microbubble was easily met.

##### Effect of the Method on Paclitaxel Encapsulation

As shown in Table 5, with PTX, there was a significant difference (*p* = 0.002) in PTX drug loading between the three groups. With the use of separate micelles (LP1), the drug load of PTX was significantly higher (*p* = 0.035) than with the LP2 microbubbles in which drugs were together in micelles. Interestingly, the PTX loading of both LP1 and LP2 was found to be significantly greater than that for PTX alone, (*p* = 0.001 and *p* = 0.031 respectively) indicating that the dual loading of LND within the microbubble shell, irrespective of the micelle loading method, significantly increased the loading of PTX. This synergistic combination of multiple drugs has been noted before in micelles [44,45,46]. Although the mechanism behind this phenomenon is not known, one could speculate that the LND intercalating in the hydrophobic tails caused a more favorable condition for PTX loading during fabrication but not vice versa. In all cases of PTX loading, as with LND, the calculated minimum required drug concentration of 2.77 µg PTX/mL microbubble was easily met in the dual-loaded platforms. These results contrast with LND loading (Table 4), which showed no increased loading upon the inclusion of PTX. 

### 3.3. In Vitro Imaging

The response of the microbubbles in a clinically relevant ultrasound beam was assessed using a tissue-mimicking flow phantom and clinical scanner. Samples of LP1-type bubbles (drugs initially in separate micelles) were chosen since, at the time of analysis, these were shown to have the highest drug encapsulation and were incorporated into the in vivo experiments. 

#### 3.3.1. Flow Phantom

**Figure 5:**
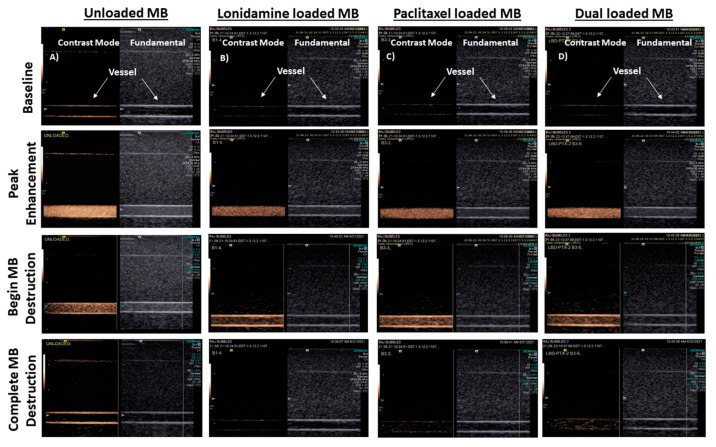
Effect of drug loading on performance in a tissue-mimicking flow phantom using SE61_O2_ microbubbles (direction of flow right to left). Each image was taken at a focal length of 4 cm, displayed in both non-linear contrast mode (left-hand image in each panel) and fundamental B-mode (right-hand image), starting with pre-injection, empty vessels taken as the baseline images. Column (**A**) Unloaded, (**B**) Lonidamine-loaded, (**C**) Paclitaxel-loaded, and (**D**) LP1dual-loaded.

As can be seen in Figure 5, all drug-loaded samples, both single and dual drug (Lp1) loaded, displayed excellent echogenicity at peak enhancement and were destroyed at higher MI during flash replenishment (MI increased from 0.12 to 0.87). The destruction was noted and imaged a few seconds after application of the higher MI, and replenishment/reperfusion was visible immediately upon resumption of non-destructive imaging. The rapid “reperfusion” opens the way to employ multiple flash sequences to deliver drugs repeatedly in vivo. These properties highlight the potential value of these agents to deliver both oxygen and more than one drug, simultaneously to areas of hypoxia in a tumor in a straightforward, safe, and site-directed, triggered, real-time fashion. This potential encompasses the reversal of both radiation resistance and multi-drug resistance.

#### 3.3.2. Stability in the Ultrasound Beam 

**Figure 6:**
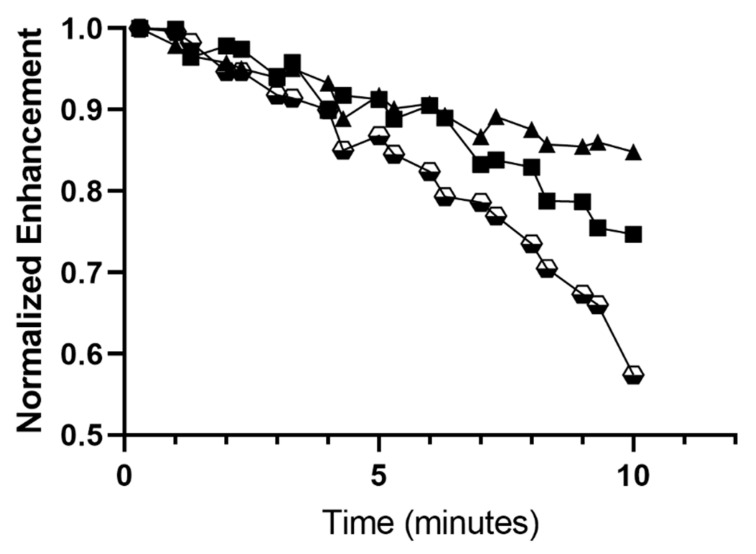
Effect of drug loading upon stability of microbubbles in an ultrasound beam at low MI. Lonidamine (-_◾_-) loaded, paclitaxel loaded (-▲-) and LP1 dual loaded (-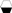-), SE61_O2_ microbubbles.

Operating at a non-destructive MI (0.12), stability curves were constructed from the flow phantom data, processing a region of interest as described above. Readings taken every 30 s were converted to normalized enhancement and the resulting plots are shown in Figure 6. The graphs indicate that the effects of ultrasound on the microbubbles, in the absence of other factors, would allow circulation with over 80% retained signal, for at least 6 min. For the dual-loaded microbubble, there appears to be a threshold at around 4 min, after which destruction, as measured by loss of dB returned to the transducer, appears to accelerate. These values are well within requirements for use of the microbubble to locate tumors and choose the optimum site to trigger destruction.

### 3.4. In Vivo Imaging

**Figure 7:**
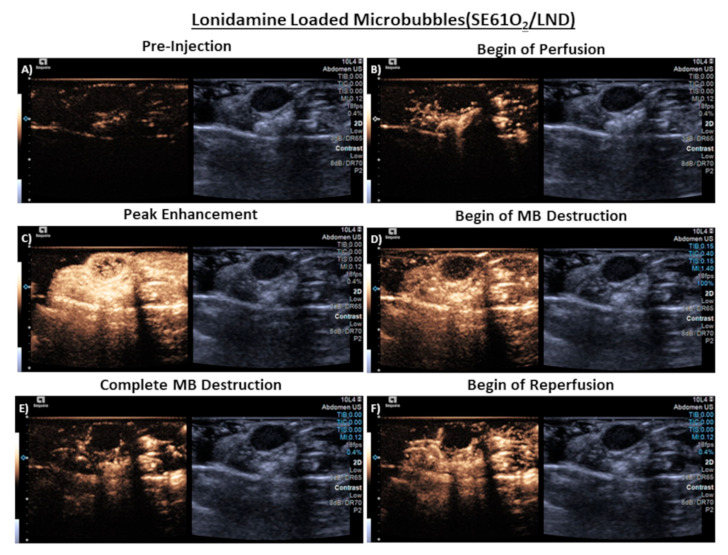
Representative ultrasound images in aythmic nude mice taken in dual B-mode/cadence pulse sequencing mode (Right/Left), imaging subcutaneous CAL27 human squamous cell carcinoma. (**A**) Pre tail vein injection of microbubbles (MB), area of tumor outlined, (**B**) Peak enhancement post injection, (**C**) Start of MB destruction, (**D**) Complete MB destruction, (**E**) Start of reperfusion, (**F**) Complete reperfusion. *Note: The reverberation artifact on the right side of some images results from the clinical transducer imaging air as a consequence of being considerably wider than the mouse torso*.

No adverse events were observed in any animal following injection of the dual-loaded microbubble, suggesting good acute tolerability of the agent. Prompt ultrasound enhancement within the tumor was evident in all three animals and lasted 3–5 min, indicating that the agent was both able to penetrate into the tumor vasculature and also remain relatively stable in vivo. The ability to locally destroy the microbubbles within the tumor vasculature was also confirmed using a series of high MI destructive pulses. An example series of images from this sequence is provided in Figure 7, showing the tumor as baseline, enhancement of the tumor vasculature during microbubble arrival, destruction of the microbubbles during higher MI pulses, and then reperfusion of the vasculature from microbubbles in systemic circulation. These findings mirror the in vitro flow phantom results and importantly indicate that the dual-loaded microbubble can be noninvasively detected within the tumor using diagnostic ultrasound and then locally destroyed. 

## 4. Conclusions

Traditional mono-chemotherapy (use of a single drug) in cancer therapy is now being greatly enhanced by exploiting the synergistic effect of delivering combinations of drugs, especially ones that act by different mechanisms. Further advances are realized by targeted drug delivery and multimodal approaches; however, all these new approaches require the development of suitable drug delivery platforms, which has ushered in the era of “combo therapy” [47]. We conclude from this proof-of-concept study that it is possible to successfully develop a multi-modal drug delivery platform that not only acts as an ultrasound contrast agent (in vitro enhancement up to 18.65 ± 0.05 dB and 18.85 ± 0.02 dB) to locate and identify the area to which drug is to be delivered but can also be triggered to deliver a payload of at least two drugs (maximum LND dose of 104.85 ± 22.87 µg LND/g microbubbles and PTX of 187.35 ± 8.38 µg PTX/g microbubbles) that it has the great advantage of possessing, together with a therapeutic gas (oxygen), in a spatiotemporal fashion. This also allows the ability to alleviate radio and chemo-resistance brought about by hypoxia. We conclude that the resulting dual-loaded microbubbles can be produced within all the constraints for intravenous injection of ultrasound contrast agents such as size and acoustic response and can achieve therapeutic drug levels. We further conclude that when using this method, greater yields are obtained if the two drugs are initially encapsulated in separate micelles and that inclusion of LND together with PTX significantly enhances the PTX but not the LND loading. Thus, we have provided a methodology to deliver two drugs in a site-directed fashion, at exactly the same location and point in time, together with O_2_ gas.

The overall strategy involved in this work lends itself to various avenues that further enhance the efficiency of drug incorporation. The original surfactant-stabilized contrast microbubble showed that stabilization can be explained by the hypothesis of opposing forces and decreased head-group repulsion in the mixed surfactant system, lending stability to the microbubble and offering an entire series of Span and Tween molecules as potential shell components. Later, we replaced Tween with TPGS, further increasing the scope of the platforms [34,48,49]. The overall strategy involved in this work lends itself to various avenues to further enhance the efficiency of drug incorporation, which is currently the major limitation. Future work will evaluate in vivo biodistribution, oxygenation, and therapeutic gain of this combinatory therapy. 

## Figures and Tables

**Table 1:** Effect of single-drug incorporation on microbubble size, concentration, and acoustic behavior.

	Diameter (µm)	Concentration × 10^9^ (Microbubbles/mL)	Maximum Enhancement (dB)	Dose at Max (MB/mL × 10^7^)
Unloaded	1.69 ± 0.10	3.30 ± 1.35	18.10 ± 0.52	4.94 ± 1.65
LND-SE61_O2_	1.74 ± 0.05	1.57 ± 0.62	18.70 ± 0.40	3.67 ± 0.91
PTX-SE61_O2_	1.68 ± 0.08	2.29 ± 0.50	18.90 ± 0.18	3.81 ± 0.66

**Table 2:** Quantification of single-drug loading.

	Added Drug (mg)	μg Drug/mL Microbubble	μg Drug/g Microbubble	µMoles Drug/g Microbubble	Encapsulation Efficiency (%)
PTX SE61_O2_	5.27 ± 0.30	4.47 ± 0.97	84.27 ± 4.39	0.0986	1.66
LND SE61_O2_	4.07 ± 0.15	4.19 ± 0.75	64.04 ± 15.62	0.0139	1.54

**Table 3:** Effect of method of dual drug incorporation on microbubble size and concentration, and acoustic behavior.

	Diameter (µm)	Concentration (Microbubbles/mL)	Maximum Enhancement (dB)	Dose at Max (MB/mL × 10^7^)
Separate Micelle incubation (LP1)	1.74 ± 0.03	2.25 ± 1.19 × 10^9^	18.63 ± 0.05	4.49 ± 0.00
Mixed Micelle incubation (LP2)	1.64 ± 0.02	1.67 ± 0.47 × 10^9^	18.85 ± 0.02	4.46 ± 0.97

**Table 4:** Effect of method of dual drug incorporation on LND encapsulation.

	µg LND/mL Microbubbles	µg LND/g Microbubble	Encapsulation Efficiency (%)
Separate TPGS micelles (LP1)	5.01 ± 0.99	104.85 ± 22.87	2.42
Mixed Incubation (LP2)	4.17 ± 0.43	87.54 ± 16.41	2.06
LND in SE61_O2_	4.19 ± 0.75	64.04 ± 15.62	1.54

**Table 5:** Effect of method of dual drug incorporation on PTX encapsulation.

	µg PTX/mL Microbubbles	µg PTX/g Microbubbles	Encapsulation Efficiency (%)
Separate TPGS micelles (LP1)	8.99 ± 0.56	187.35 ± 8.38	3.48
Mixed Incubation (LP2)	6.50 ± 1.01	136.51 ± 30.66	2.56
PTX in SE61_O2_	4.47 ± 0.97	84.29 ± 4.39	1.66

## Data Availability

Data will be made upon request.

## Supplementary Materials

The following supporting information can be downloaded at: https://www.mdpi.com/article/10.3390/polym14081568/s1, Figure S1: Representative size distribution profiles of SE61_O2_ microbubbles. Unloaded (-●-), Single loaded lonidamine (-◾-), paclitaxel (-▲-), dual loaded LP1 (-◆-), and LP2 (-✮-); Figure S2: Light Microscopy images of the SE61_O2_ MBs taken under 40× with 1.6× camera magnification, 20 μm size bar shown for reference. (A) Unloaded SE61_O2_ (B) LND-loaded SE61_O2_ (C) PTX loaded SE61_O2_. Sizes noted by selected bubbles processes using the Olympus cellSens Standard software (Olympus Corporation, Tokyo, Japan); Figure S3: Light Microscopy images of dual loaded SE61_O2_ microbubbles taken under 40× with 1.6× camera magnification, 20 μm size bar shown for reference (A) LP1 (B) LP2. Sizes noted by selected bubbles processes using the Olympus cellSens Standard software (Olympus Corporation, Tokyo, Japan).
