# Supplementary material for: Development of a Dual Drug-Loaded, Surfactant-Stabilized Contrast Agent Containing Oxygen
Source: Polymers (Basel). 2022 Apr 12;14(8):1568. doi: 10.3390/polym14081568 (PMC9027498; doi:10.3390/polym14081568)
Supplement: Supplementary file 1 [file polymers-14-01568-s001.zip › polymers-1649518-supplementary.pdf]

Supplemental

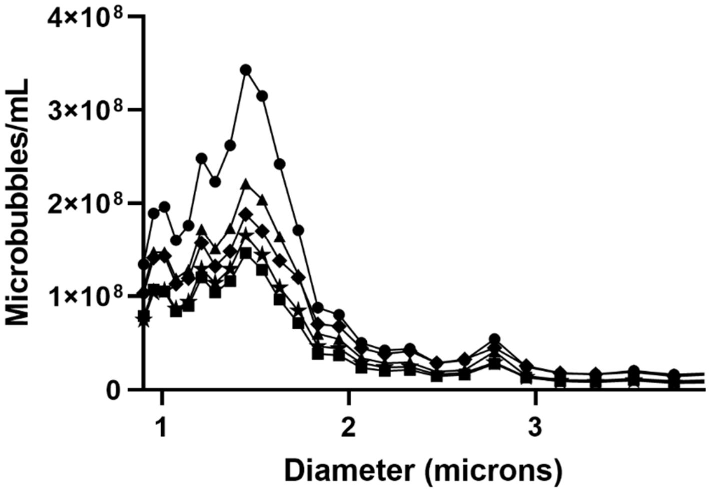

Figure. S1, Representative size distribution profiles of SE61O2 microbubbles. Unloaded (-●-), Single loaded lonidamine (-▪-), paclitaxel (-▲-), dual loaded LP1 (-◆-), and LP2 (-✯-)

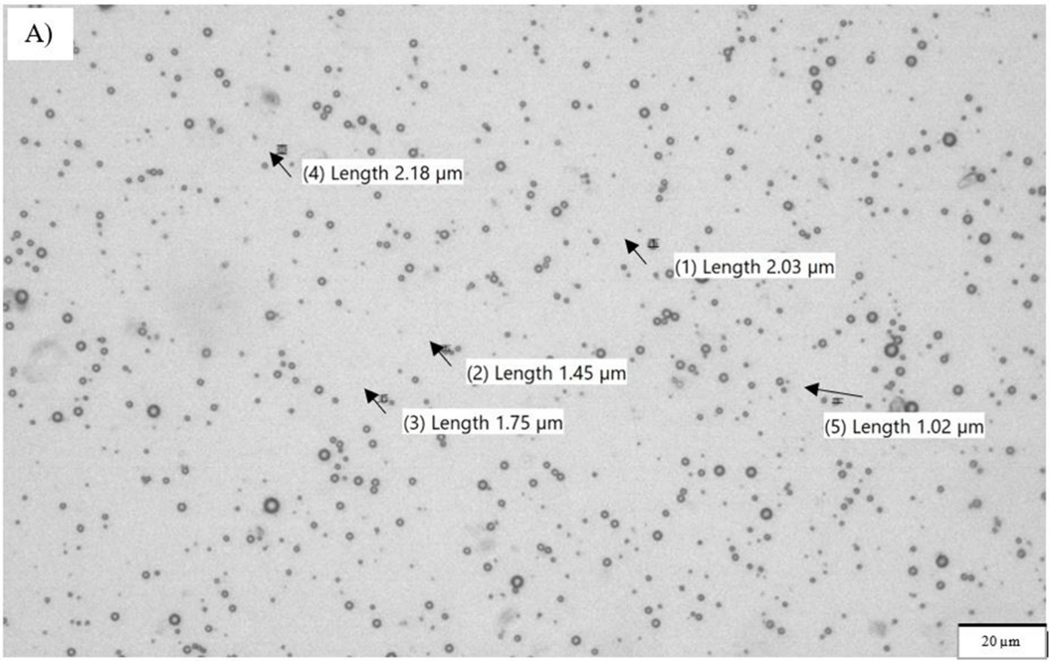
A)
(4) Length 2.18 µm
(1) Length 2.03 µm
(2) Length 1.45 µm
(3) Length 1.75 µm
(5) Length 1.02 µm
20 µm

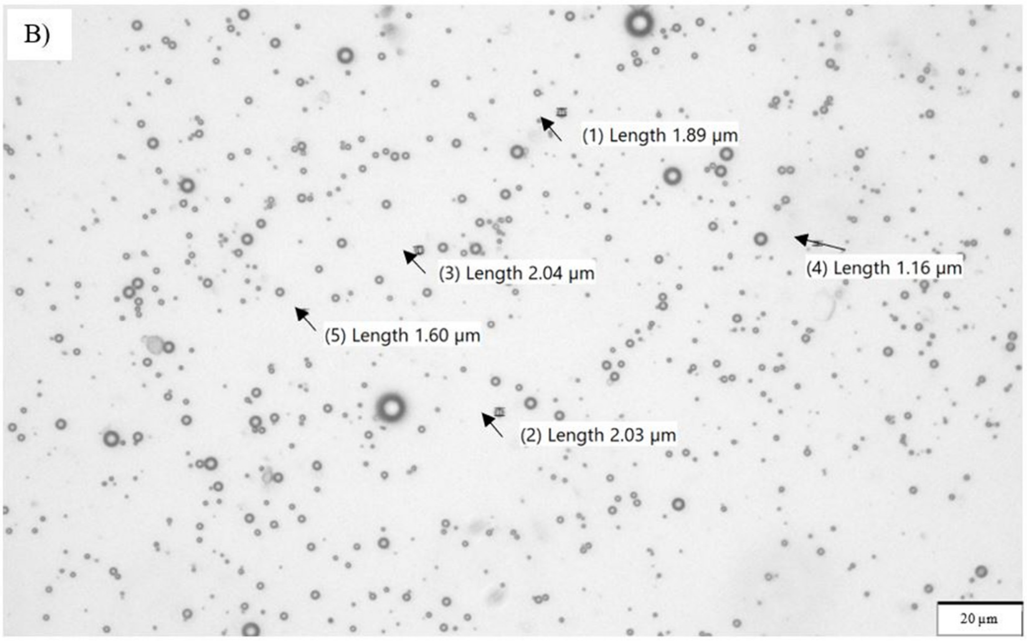
B)
(1) Length 1.89 µm
(3) Length 2.04 µm
(4) Length 1.16 µm
(5) Length 1.60 µm
(2) Length 2.03 µm
20 µm

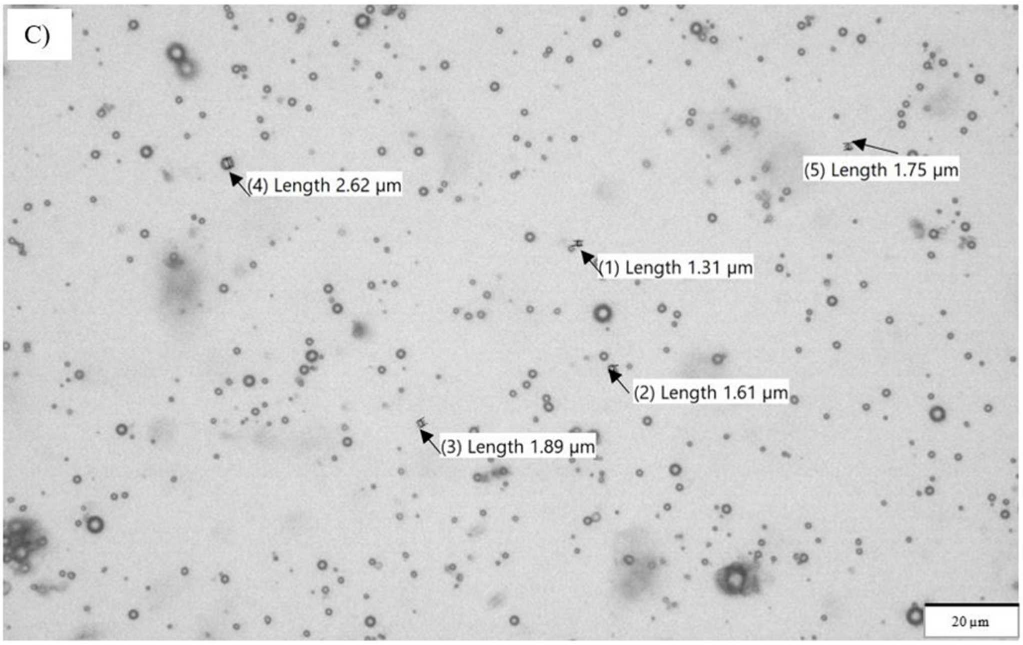

Figure S2. Light Microscopy images of the SE61$_{O2}$ microbubbles taken under 40x with 1.6x camera magnification, 20 µm size bar shown for reference. A) unloaded B) Lonidamine-loaded C) Paclitaxel -loaded. Sizes noted by selected bubbles processes using the Olympus cellSens Standard software (Olympus Corporation, Tokyo, Japan).

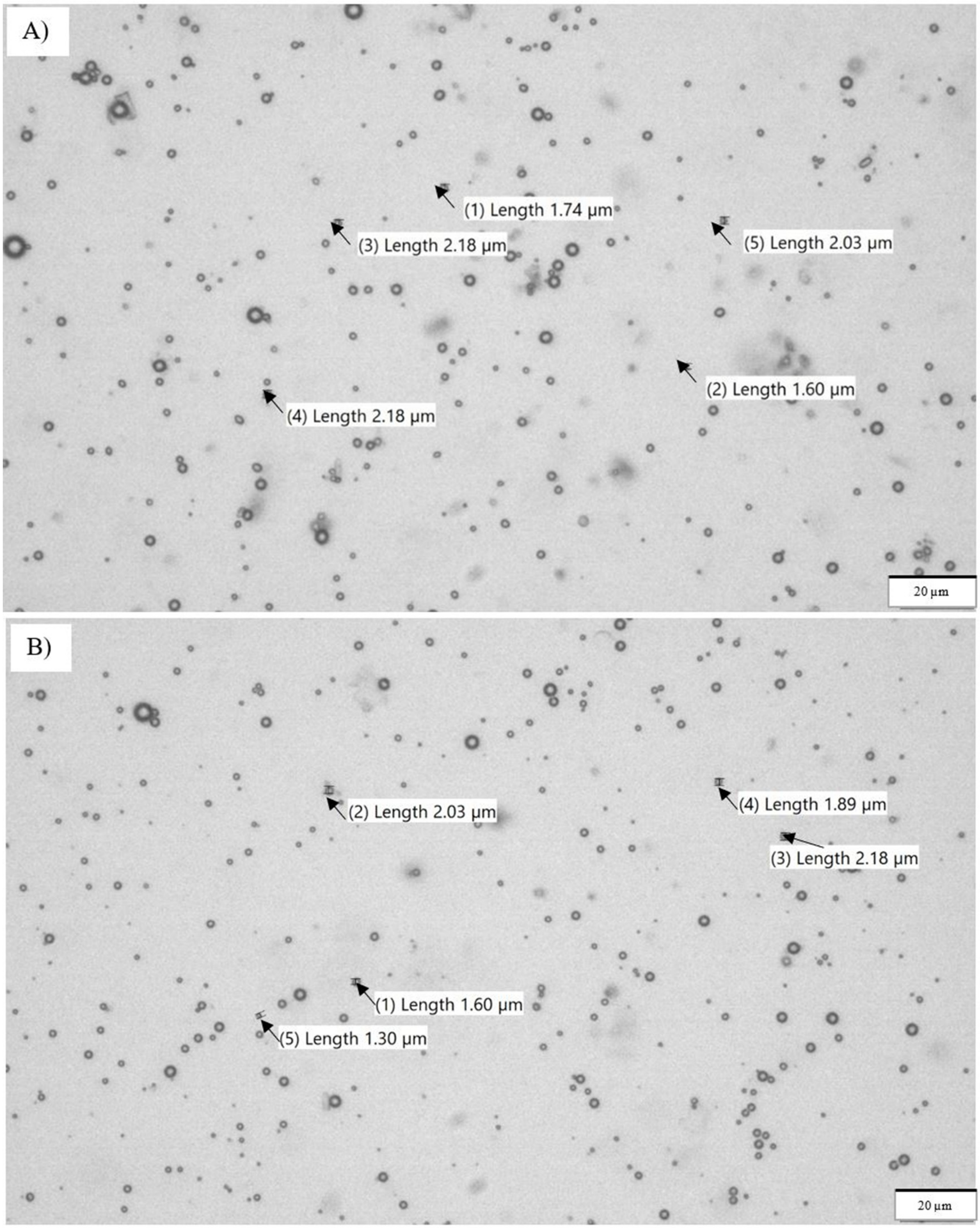

Figure S3. Light Microscopy images of the dual loaded SE61$_{O2}$ microbubbles taken under 40x with 1.6x camera magnification, 20 µm size bar shown for reference. A) LP1, B) LP2. Sizes noted by selected bubbles processes using the Olympus cellSens Standard software (Olympus Corporation, Tokyo, Japan).

List of figures for Supplemental

Figure S1. Representative size distribution profiles of SE61$_{O2}$ microbubbles. Unloaded (-●-), Single loaded lonidamine (-▪-), paclitaxel (-▲-), dual loaded LP1 (-◆-), and LP2 (-✯-)

Figure S2. Light Microscopy images of the SE61$_{O2}$ MBs taken under 40x with 1.6x camera magnification, 20 µm size bar shown for reference. A) Unloaded SE61 $_{O2}$ B) LND-loaded SE61$_{O2}$ C) PTX loaded SE61 $_{O2}$. Sizes noted by selected bubbles processes using the Olympus cellSens Standard software (Olympus Corporation, Tokyo, Japan)

Figure S3. Light Microscopy images of dual loaded SE61$_{O2}$ microbubbles taken under 40x with 1.6x camera magnification, 20 µm size bar shown for reference A) LP1 B) LP2. Sizes noted by selected bubbles processes using the Olympus cellSens Standard software (Olympus Corporation, Tokyo, Japan).
